# On the origins of transport inefficiencies in mesoscopic networks

**DOI:** 10.1038/s41598-018-21250-y

**Published:** 2018-02-14

**Authors:** Sébastien Toussaint, Frederico Martins, Sébastien Faniel, Marco G. Pala, Ludovic Desplanque, Xavier Wallart, Hermann Sellier, Serge Huant, Vincent Bayot, Benoit Hackens

**Affiliations:** 1 Université catholique de Louvain, Institute of Condensed Matter and Nanosciences (IMCN/NAPS), Louvain-la-Neuve, B-1348 Belgium; 2 Centre de Nanosciences et de Nanotechnologies, Université Paris-Sud, Université Paris-Saclay, CNRS, Orsay, F-91405 France; 3 Université Lille, CNRS, Centrale Lille, ISEN, Univ. Valenciennes, UMR 8520 - IEMN, Lille, F-59000 France; 4 Institut Néel, Université Grenoble Alpes and CNRS, Grenoble, F-38042 France

## Abstract

A counter-intuitive behavior analogous to the Braess paradox is encountered in a two-terminal mesoscopic network patterned in a two-dimensional electron system (2DES). Decreasing locally the electron density of one channel of the network paradoxically leads to an increased network electrical conductance. Our low temperature scanning gate microscopy experiments reveal different occurrences of such puzzling conductance variations, thanks to tip-induced localized modifications of electron flow throughout the network’s channels in the ballistic and coherent regime of transport. The robustness of the puzzling behavior is inspected by varying the global 2DES density, magnetic field and the tip-surface distance. Depending on the overall 2DES density, we show that either Coulomb Blockade resonances due to disorder-induced localized states or Fabry-Perot interferences tuned by the tip-induced electrostatic perturbation are at the origin of transport inefficiencies in the network, which are lifted when gradually closing one channel of the network with the tip.

## Introduction

The suppression of an axis in a transport network can surprisingly improve the overall network performances. This counterintuitive behavior - initially highlighted in road networks - is known as the Braess paradox^1^, formalized in the framework of game theory. In short, the combination of selfish behaviors and non-linear response of traffic roads paradoxically increase transit time when new roads are added. The Braess paradox was later evidenced in electrical, hydraulics and mechanical networks^2^. In the latter cases, selfishness is obviously not at stake, but non-linear response is. In the linear classical regime, however, no physical system is expected to exhibit such paradox. The question is then: Is quantum mechanics a game changer?

This question was first addressed by means of simulations and experiments on an electronic network^3^. Downscaling such a network, one can reach the mesoscopic regime of transport where quantum behaviors such as energy quantization, quantum wave interferences and single charge effects become prominent. Performing a transport experiment on a mesoscopic network fabricated from InGaAs/InAlAs heterostructure, several authors of the present work revealed a behavior analogous to the Braess paradox^3^ : the network electrical conductance *G* was found to increase under depletion of its central axis. However, neither selfishness nor non-linear response can be invoked to explain what is - at first sight - perceived as a striking anomaly. While simulations based on the Keldysh-Green formalism predict such paradoxical behavior^4^, its possible physical origins are not yet experimentally clarified. Simulations by Sousa *et al*.^5^ and Macucci *et al*.^6^ both conclude that different scenarios based on electron wave interferences can indeed yield conductance increase upon transport axis closures. The questions are now: can we pinpoint experimental conditions where interferences induce paradoxical conductance improvement, and, are there other physical mechanisms able to induce such phenomenon?

The aim of this paper is to address these questions. For this purpose we perform conductance measurements on a mesoscopic network while tuning the local electron density by means of a moving nanoscopic scanning gate. We first confirm the existence of a paradoxical behavior in the quantum regime of transport, and then identify two distinct mechanisms that can be at its origin.

**Figure 1:**
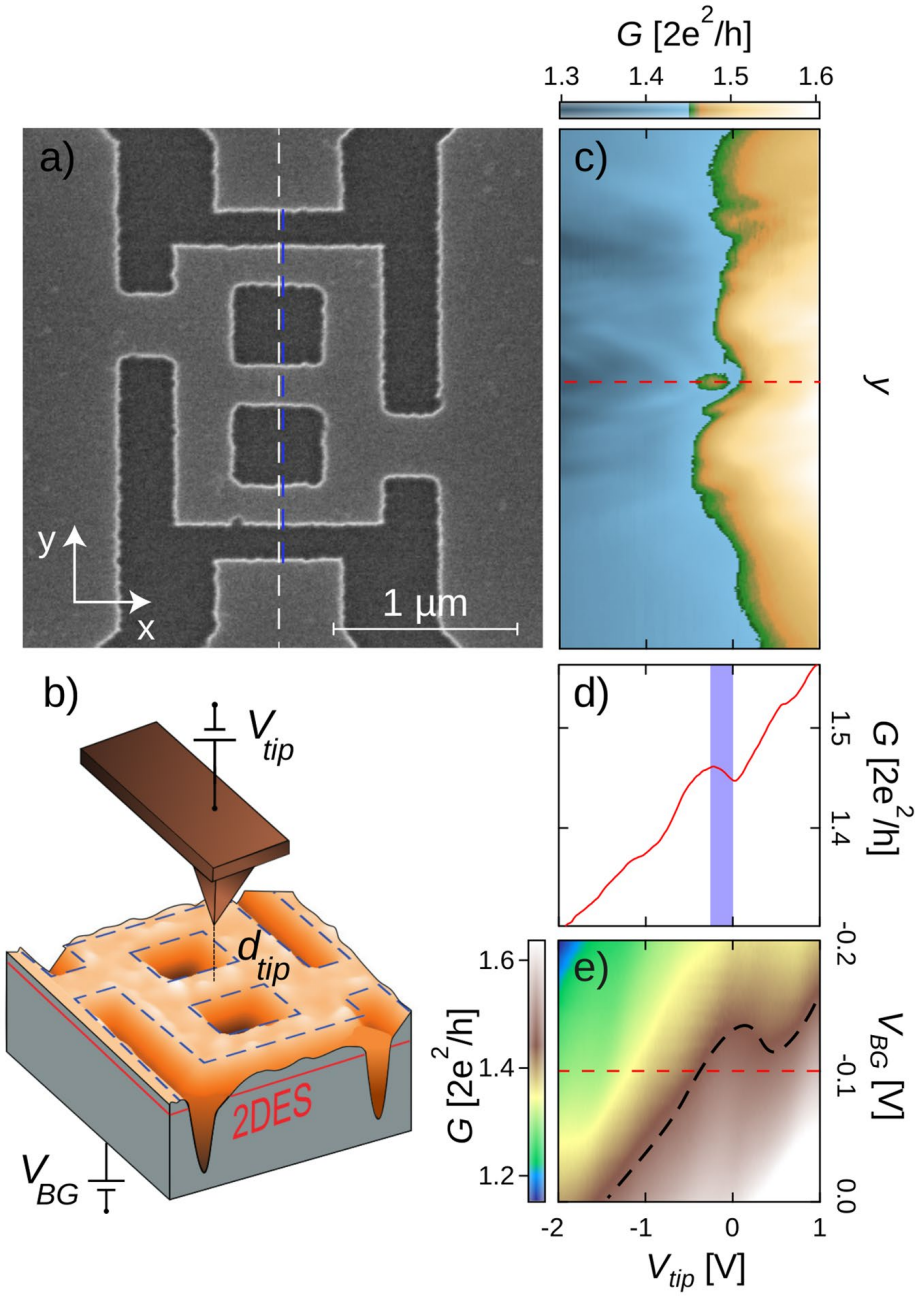
(**a**) Scanning electron micrograph of the network whose conductance (*G*) is measured. (**b**) AFM topography of the network embedded in an artist’s view of the tip-network system. The different parameters present in an SGM experiment are shown: the tip potential (*V*_*tip*_), the tip-sample surface distance (*d*_*tip*_) and the backgate potential (*V*_*BG*_). (**c**) Conductance measurement as a function of *V*_*tip*_ with the tip scanned along the dashed white line in (**a**), for *d*_*tip*_ = 80 nm and *V*_*BG*_ = −0.1 V. The vertical axes of (**a**) and (**c**) are matched. Note that the same data is presented in Supplementary Fig. 1a with a different color scale, and Supplementary Fig. 1b displays a mapping of $\frac{{\rm{d}}G}{{\rm{d}}{V}_{tip}}|{V}_{BG}$ calculated from data in Fig. 1c. (**d**) *G* measurement along the dashed red line in (**c**) and (**e**). Note that the anomaly in (**c**) and (**d**) occurs when the tip is right above the central branch of the network. (**e**) *G* mapping as a function of *V*_*tip*_ and *V*_*BG*_ with the tip located 80 nm above the middle of the central channel. The black dashed line follows an iso-conductance line.

We study the sample shown in Fig. 1a, similar in size and shape to the one studied in ref.^3^ and patterned using electron beam lithography and wet etching in an InGaAs/InAlAs heterostructure. A two- dimensional electron system (2DES) is confined 45 nm below the surface of the heterostructure that was grown by molecular beam epitaxy on a degenerately- doped InP substrate (InP n+) serving as a backgate to tune the global 2DES density. The experiments were performed at *T* = 4.2 K where the electron density *n*_2*D*_ - obtained by analyzing Shubnikov-de Haas (SdH) oscillations on a neighboring Hall bar - varies from 6 to 8.4 × 10^11^ cm^−2^ as the backgate potential *V*_*BG*_ goes from 2 to 4 V. Simultaneously the mobility varies from 9 to 11 $\frac{{{\rm{m}}}^{2}}{{\rm{Vs}}}$. The device conductance *G* is measured using a low-frequency (28.5 Hz) standard lock-in technique, with the source-drain voltage across the device always being less than $\frac{{k}_{B}T}{e}$ to ensure quasi-equilibrium conditions.

In order to selectively perturb transport in the network channels, we used the scanning gate microscopy (SGM)^7,8^ technique that allows us to locally change the electrostatic potential landscape thanks to a polarised conductive atomic force microscope tip of apex radius smaller than 20 nm. The tip is attached to a tuning fork used as a force sensor to map the topography (see Fig. 1b) of the sample and to precisely determine the relative tip-network position^9^. The conducting AFM tip is then withdrawn at a distance *d*_*tip*_ from the sample surface - typically a few tens of nanometers - and scanned in a plane parallel to the 2DES. All the following parameters, the tip position *x*_*tip*_, *y*_*tip*_, height *d*_*tip*_ and polarization *V*_*tip*_ change the electrostatic potential landscape within the network and lead to variations of the device electrical conductance. The conductance is mapped as a function of *x*_*tip*_, *y*_*tip*_, *V*_*BG*_, *V*_*tip*_, *d*_*tip*_ and magnetic field *B*, which will generate all the data presented hereafter.

## Results and Discussion

**Figure 2:**
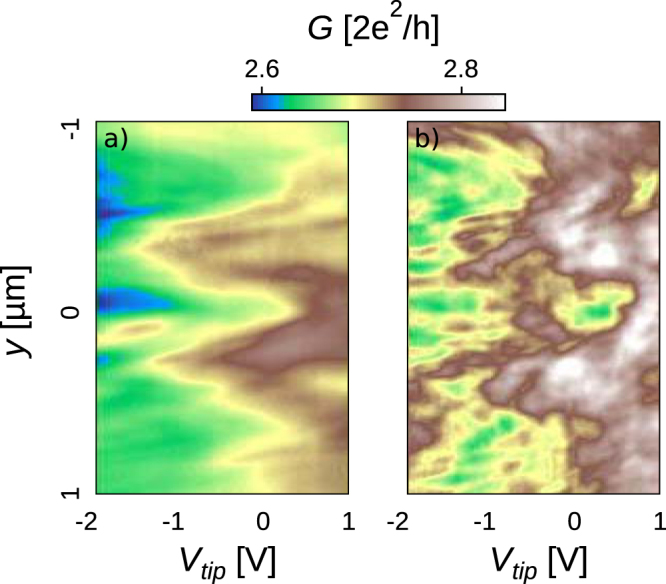
(**a**) Mapping of the SGM linescan along the blue dashed line in Fig. 1a, as a function of *V*_*tip*_ at 4.2 K, exhibiting an anomalous and robust increase of conductance when the tip scans above the central arm, for −2 *V* < *V*_*tip*_ < −1 *V*. (**b**) The same mapping is obtained at 50 mK. *V*_*BG*_ = 0.8 *V* for both mappings. Note that the mappings of the derivative of data in Fig. 2b are presented in Supplementary Fig. 1c and d (*i.e*. derivative of G with respect to *V*_*tip*_ and *y*, respectively).

As a first experiment, we measure the device conductance as the polarized tip is scanned along the ashed line on Fig. 1a, 80 nm above the sample surface. As *V*_*tip*_ becomes more negative, we observe an anomalous conductance behavior when the tip is located above the central channel (along the red curve in Fig. 1d). On a particular *V*_*tip*_ domain, the conductance paradoxically rises as the negative *V*_*tip*_ progressively depletes the 2DES underneath the tip. Figure 1c shows that this peculiar behavior occurs only when the tip is located right above the central branch, as reported in ref.^3^. Beyond reproducing comparable results, we now investigate their robustness while varying the average charge carrier density in the whole network. This is done in Fig. 1e where the conductance is mapped in the *V*_*tip*_ − *V*_*BG*_ plane with the tip located above the middle of the central branch. The black-dotted isoconductance line gives a different perspective to the anomalous behavior we are focused on. Indeed, the anomaly could either be evidenced by a negative $\frac{{\rm{d}}G}{{\rm{d}}{V}_{tip}}|{V}_{BG}$ or by a negative $\frac{{\rm{d}}G}{{\rm{d}}{V}_{BG}}{|}_{{V}_{tip}}$. One novelty revealed by the present experiment is that the anomalous behaviour can be observed both for negative or positive tip potentials, which was not expected from earlier works^3^. For instance, Fig. 1e reveals that the anomaly drifts towards positive *V*_*tip*_ when the 2DES density is lowered (*V*_*BG*_ < 0). This fact is indeed worth emphasizing as it shows that depletion is not necessarily a key ingredient for the observed anomaly. Figure 2a shows a line scan analogous to Fig. 1c, but for a positive value of *V*_*BG*_. Consistently with the trend observed in Fig. 1, the anomalous bump is now observed at more negative values of *V*_*tip*_, and remains located above the central branch.

Observing the evolution of the amplitude of transport phenomena with temperature is often key to pinpoint their origin. Indeed, the characteristic times governing transport mechanisms follow different temperature dependences. For exemple, the phase coherence time *τ*_*ϕ*_ increases in most cases when lowering the temperature, which leads to an enhanced amplitude of electron interference phenomena. In this experiment, we surprisingly observe strong qualitative changes when lowering the temperature: in the linescan data obtained at 50 mK (Fig. 2b) in the same conditions as Fig. 2a, the contrast is dominated by strong conductance fluctuations, and the anomalous behaviour observed at 4.2 K over the central branch is no longer visible. The large amplitude random fluctuations evidenced at the lowest temperature are a hallmark of Universal Conductance Fluctuations (UCFs), a phenomenon stemming from interferences between a large number of different semiclassical electron paths^10^. The enhancement of UCFs contributions is consistent with the increased phase coherence time. However, if the anomalous bump of Fig. 2a was governed by the same phase coherence time as the one determining the UCFs’ amplitude, it should be no more visible than the random fluctuations attributed to UCFs in Fig. 2b. Hence, data in Fig. 2 advocate against UCFs as the origin of the anomalous behaviour. In the remainder of this paper, we will focus on the data collected at 4.2 K, where the anomalous behavour is more clearly visible, and we will come back to the interpretation of data in Fig. 2 at the end of the discussion. Note also that additional thermal cycles have been performed and confirmed the robustness of the effect.

**Figure 3:**
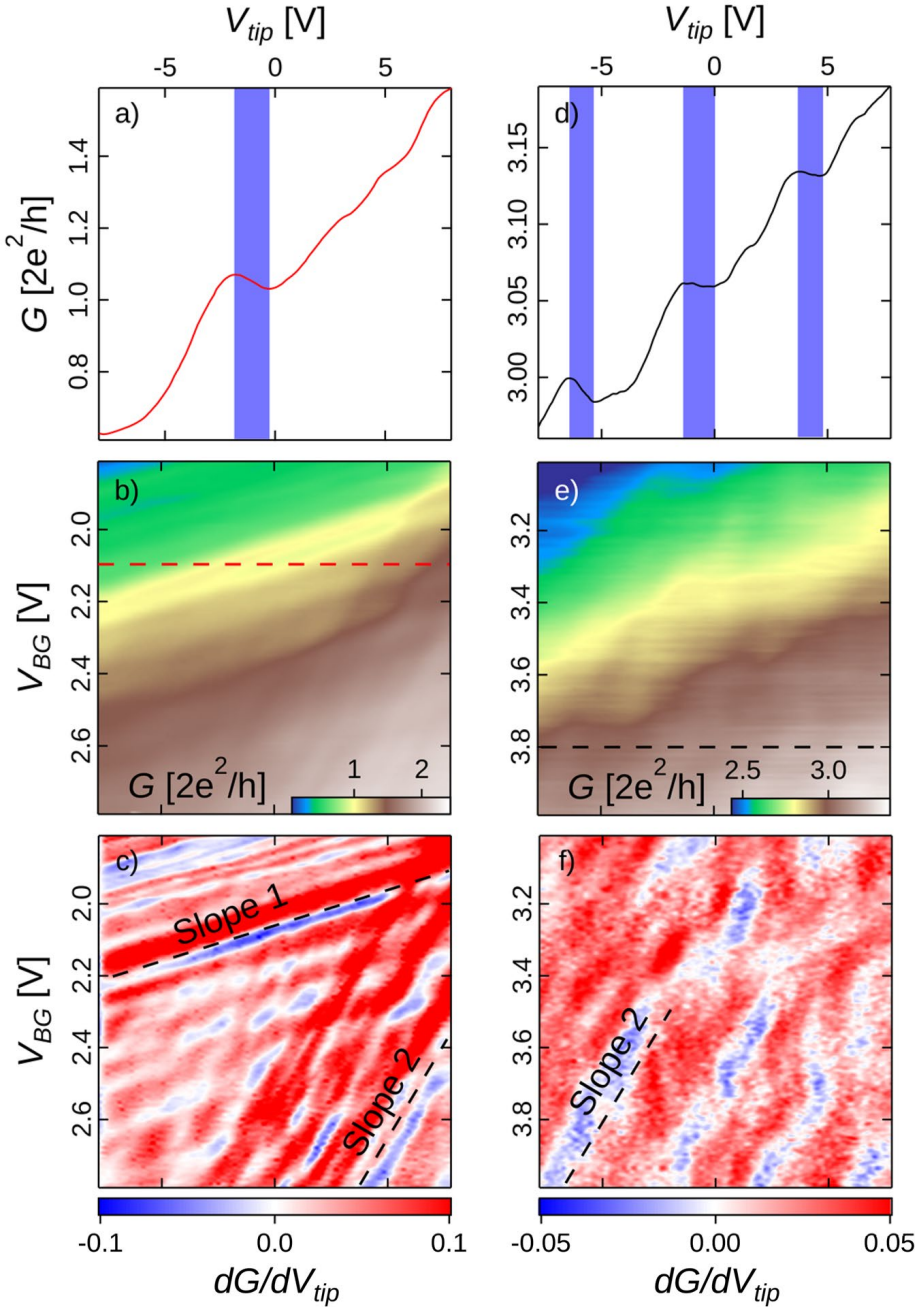
(**a**) and (**d**) Conductance measurement when varying *V*_*tip*_ along the dashed lines in (**b**) and (**e**) corresponding to different ranges of electron densities. (**b**) and (**e**) Conductance measurement as *V*_*tip*_ and *V*_*BG*_ are swept with *d*_*tip*_ = 50 nm for (**b**) and 100 nm for (**e**) above the middle of the central channel (see Fig. 1a). (**c**) and (**f**) present the derivative of *G* with respect to *V*_*tip*_ at constant *V*_*BG*_ associated to the *G* mappings presented in (**b**) and (**e**). All *V*_*tip*_ horizontal axis are matched.

We now investigate the conditions required for the emergence of anomalous conductance domains when sweeping both *V*_*tip*_ and *V*_*BG*_ with the tip sitting at a constant distance above the middle of the central channel. The result is presented in Fig. 3 that provides a deeper insight in the density dependence of the studied behavior. The two sets of data differ by the doubling of *d*_*tip*_ and a different range in *V*_*BG*_, and hence 2DES density ranges. From the conductance maps shown in Fig. 3b and e, we extracted two conductance plots at two specific *V*_*BG*_ (Fig. 3a and d) where the targeted anomalies are emphasized by blue domains. The detection of paradoxical conductance variations in the conductance maps is made easier by plotting $\frac{{\rm{d}}G}{{\rm{d}}{V}_{tip}}|{V}_{BG}$ as a function of *V*_*BG*_ in Fig. 3c and f, since the signature of the paradox is a negative sign in this derivative (blue color).

A major piece of information resides in Fig. 3c and f, where the presence of two sets of stripes - with significantly different slopes - is the main feature of the data. The phenomenology of stripes, i.e. a gradual and regular shift of periodic oscillations with respect to *V*_*tip*_ and *V*_*BG*_, does not correspond at all to that of UCFs, which are random by nature^10^, and therefore undergo a chaotic evolution when external parameters such a gate voltage and a magnetic field are varied. As already mentioned, UCFs are necessarily present since transport is in the coherent regime - as evidenced by the Aharanov-Bohm (AB) oscillations^11^ and *l*_*ϕ*_ estimates discussed later in the paper - but, at 4.2 K, they are dominated by two other effects that we will now investigate. In contrast, when temperature is lowered down to 50 mK, UCFs become dominant, as shown on Fig. 2b. This is another clear sign that the phenomena revealed in Fig. 3 are fundamentally different from UCFs.

The so-labelled slopes 1 and 2 characterize the presence of conductance anomalies which appear in blue in Fig. 3. Slope 1 characterizes isoconductance lines in Fig. 3b which are mainly governed by the backgate which tunes the overall electron density in the network. Mainly, but not completely, which means that the tip does not only have a local effect on the electron potential in the network, but also affects the global electron density. Expressed in terms of coupling capacitance, that to the back gate is 50 times larger than that of the tip. In short, slope 1 anomalies are directly related to the overall electron density as mainly tuned by *V*_*BG*_, or marginally by *V*_*tip*_. By the way, we also note that slope 1 features progressively leave the floor to slope 2 at higher conductances *i.e*. higher electron densities. The latter point may be reminiscent of single charge effects, but we will need SGM imaging data (presented later) to tell more about the physical mechanism behind slope 1.

Slope 2, on the other hand, does not follow isoconductance profiles and is much steeper than slope 1, which clearly points towards a mechanism where the local impact of the tip is prominent, if not dominant.

At this stage, it seems clear that slope 1 and 2 sign two distinct mechanisms and this will be further evidenced by SGM imaging data. Slope 2 is not vertical, which means that the second mechanism is affected by the local potential in the central arm, but also depends on the overall density in the network. Slope 2 also spreads equally in both positive and negative *V*_*tip*_ and remains unaffected on a large range of conductance. Slope 2 additionally remains roughly unchanged in Fig. 3c and f.

In order to differentiate the two mechanisms, we use the ability of the SGM technique to yield real space insight into the network. The proximity of a polarised tip perturbs the potential landscape along the electron flow, which affects the macroscopic value of *G*. The measurement of *G*(*x*_*tip*_, *y*_*tip*_) when scanning the tip in a plane - at a few tens of nanometers - parallel to the sample surface allows to get local information in the form of SGM conductance maps.

**Figure 4:**
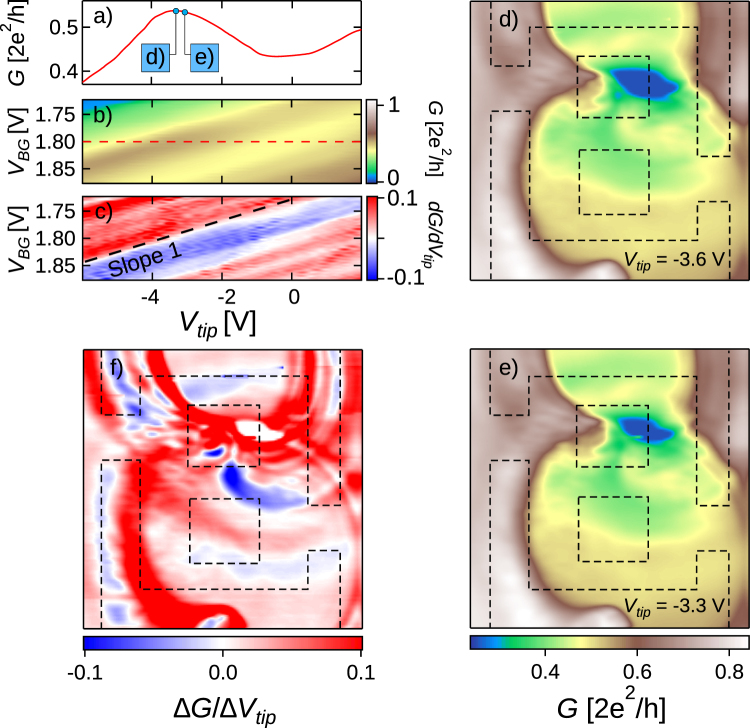
(**a**) Conductance measurement extracted from (**b**) (red dashed line) (**b**) Conductance measurement as *V*_*tip*_ and *V*_*BG*_ are swept with the tip located 50 nm above the middle of the central channel (see Fig. 1a). (**c**) Derivative of *G* with respect to *V*_*tip*_ at constant *V*_*BG*_ corresponding to the *G* mapping in (**b**). (**d**) And (**e**) Scanning gate microscopy conductance maps of the network realised for closeby *V*_*tip*_ for the tip scanned in a plane parallel to the surface and located 50 nm above it and with *V*_*BG*_ = 1.8 V. (**f**) Subtraction of the two SGM conductance mappings showed in (**d**) and (**e**).

Figure 4d and e show SGM maps measured in the range where slope 1 is dominant (Fig. 4a–c). Taken at the two closeby values of *V*_*tip*_ pointed on Fig. 4a, the two SGM images (Fig. 4d and e) have clear resemblances. In particular, we note a strong spot that marks a position where the tip brings the conductance close to zero. The mechanism behind slope 1 should thus be able to “close” all 3 arms while the tip sits above one specific region (note that in this experiment, we do not have precise information on the conductance of each parallel arm within the device). To go further in our understanding of Fig. 4d and e, it is useful to realise a subtraction of the two SGM images (Fig. 4f) as it highlights the small variations atop of a strong varying background. This data allows to identify sets of concentric fringes originating from different locations in the network, and is clearly reminiscent of the data reported by Liu *et al*. in a similar device^12^, as well as SGM data obtained on patterned quantum dots in the Coulomb blockade regime^13^ (note that the evolution of the concentric fringe pattern is shown as a function of *V*_*tip*_ in Fig. S2c–j). This observation tells us that the mechanism behind slope 1 is most probably related to single charge effects^14–17^. Even though the low conductance spot is not atop the central arm, the long-range contribution of the tip to the electron potential landscape produces strong conductance variations when *V*_*tip*_ varies while the tip sits above the central arm. The fact that the so-called “slope 1” anomalies locate along iso-conductance lines is also consistent with charging effects which are directly related to the long-range potential perturbation in the network. The suppression of slope 1 features at higher densities also advocates for single charging effects of disorder-induced quantum dots (QDs), which naturally disappear as the bottom of the conduction band is lowered. In our device, such QDs are therefore present along all three branches. The presence of QDs within a 2DES drastically affects the conductance of the device whenever transport occurs through the QD regions and when the coincidence of the dots energy levels and the Fermi energy is tuned by an external gate. Here, varying the backgate or the tip voltage changes the number of trapped charges within each QD, which induces Coulomb blockade (CB) dips in the device conductance. Since these CB dips are located on concentric fringes in SGM images, without particular spatial symmetry, one way to practically see a clear signature of Coulomb blockade is to subtract two SGM images realised for closeby tip voltages. This is exactly the purpose filled by Fig. 4f.

As evidenced in ref.^12^, discharging events of disorder-induced QD occur when the negatively biased tip approaches the dot location. Pinpointing the actual dot locations is not straightforward. Indeed both the tip geometry and complex anisotropic screening in the network lead to a tip induced potential perturbation difficult to evaluate. A priori, the potential perturbation and the QD present no particular symmetries. Nevertheless, QDs locations can be roughly estimated within the network near the centers of the concentric fringes presented in Fig. 4f, *i.e*. in the top branch and near the right exit of the network. Figure 4f also reveals that few conductance variations are observed inside the innermost fringes. This can be attributed either to a completely emptied dot or to potential barriers becoming too high for significant tunneling events to occur. This innermost region naturally expands as the tip is polarised more negatively^12^. Visually this leads to the expansion of concentric fringes as closeby-*V*_*tip*_ SGM images are subtracted for more negative tip potential pairs. This expansion is at the heart of the non monotonous conductance behavior observed in Fig. 4a where the tip is standing above the middle of the central channel. Indeed, as demonstrated in Supplementary Figure S2, a white concentric fringe (evidenced in Fig. 4f), corresponding to $\frac{{\rm{d}}G}{{\rm{d}}{V}_{tip}}=0$, crosses the central branch and a clear correspondence is established between the conductance maximum in Fig. 4a and the movement of this fringe over the middle of the central channel. In this picture, the average network density is the main parameter for the slope 1-mechanism, while small local potential variations due to the tip induce the contrast in G-maps. Consistently with Fig. 3c, concentric fringes are also observed in SGM mappings realised for closeby-*V*_*BG*_ mappings at constant *V*_*tip*_.

At this point, we revealed a first mechanism, CB, explaining the anomalous behaviour. Interestingly, we can strongly attenuate CB oscillations by increasing the global electron density. As shown on Fig. 3f, slope 1 - related features vanish at higher density, which means that disorder-induced localized states do not rule conductance in the network any more. Consistently, no concentric fringes can be observed for closeby-*V*_*tip*_ SGM images realised at higher density (see Supplementary Figure S3).

**Figure 5:**
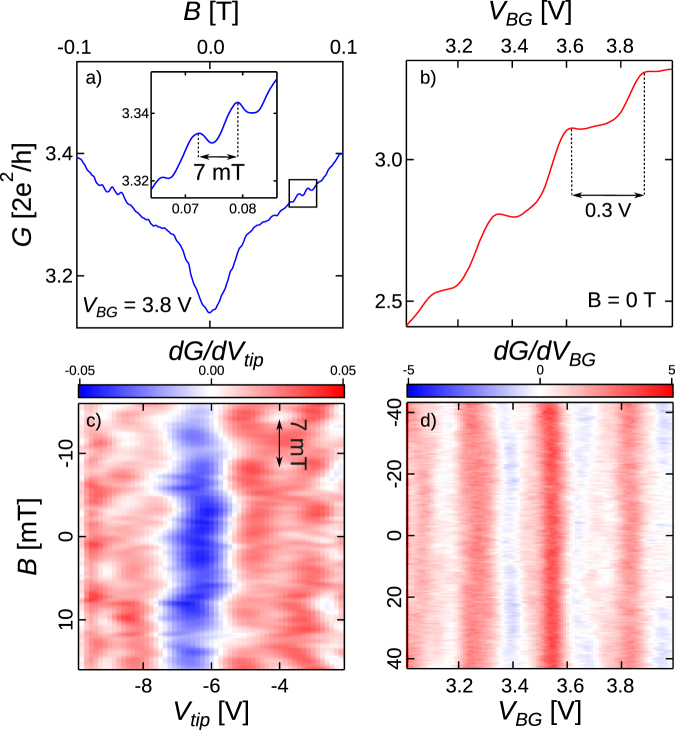
(**a**) and (**b**) Network’s conductance as (**a**) the magnetic field (*B*) and (**b**) the back-gate potential (*V*_*BG*_) are swept in absence of nearby AFM tip. The inset of Fig. 5a highlights the AB oscillations whose period are 7 mT. (**c**) Derivative of *G* with respect to *V*_*tip*_ vs *B* and *V*_*tip*_ with a tip polarized above the middle of the central branch for *d*_*tip*_ = 50 nm with *V*_*BG*_ = 3.8 V. (**d**) Derivative of *G* with respect to *V*_*BG*_ vs *B* and *V*_*BG*_ with no tip in the network’s vicinity.

We now turn to slope 2 that originates from a qualitatively different mechanism. At this point one has to recall what qualitatively changes when rising the 2DES density. In addition to a stronger tip potential screening, the amplitude of coherent effects rises. The latter is confirmed by magneto-conductance data taken at different densities (from *V*_*BG*_ = 2 to 4 V) with no tip in the network vicinity. One of these curves is presented in Fig. 5a. Sweeping a magnetic field orthogonal to the 2DES clearly reveals *G*-oscillations with a period of 7 mT (see inset of Fig. 5a). This B-period corresponds to the AB period for electrons circulating around an antidot present in the network (see Fig. 1a)^11,18^. Indeed, the 7mT-oscillations amplitude increases strongly and then saturates as *V*_*BG*_ goes from 2 to 4 V, with a threshold near 2.2 V (see Supplementary Figure S4 where a detailed analysis of the AB oscillations is presented) which is also the point where slope 1 leaves the floor to slope 2. The picture is now even clearer as one expects AB effects to be much attenuated or absent in the presence of the potential barriers needed for CB. The transition to the AB regime occurs in the range 2.2 < *V*_*BG*_ < 3.2 V, beyond which the amplitude saturates as the CB potential barriers are no longer significant. The presence of AB and even Altshuler-Aronov-Spivak (AAS) oscillations^19^ implies that a coherent mechanism could be at play in the case of slope 2 anomalies. In addition, from the analysis of the autocorrelation function of magnetoconductance traces^20,21^, we extracted a coherence length ${l}_{\varphi } \sim 1\,\mu m$ comparable to the size of the network in the *V*_*BG*_ range where slope 2 related features are observable.

In this framework, one might suggest that the tip potential perturbation could modulate the interferences in the network through the electrostatic AB effect. However, the anomalous conductance variations are one order of magnitude larger in amplitude than the observed magnetic AB effect. Indeed, in the electron density range between 7 and 8.4 × 10^11^ cm^−2^ where AB effects are fully developed (*i.e. V*_*BG*_ between 3 and 4 V), the RMS amplitude of the anomalous effect is on average $\sim 1.5\times \,{10}^{-2}{G}_{0}$ while the magnetic AB amplitude is < 2 × 10^−3^*G*_0_ (with G_0_ = 2e^2^/h). Note that measurements on quantum rings realised in similar heterostructures revealed that the electrostatic and magnetic AB effect have equal amplitudes^22^. This is a strong indication that the anomalous phenomenon is not compatible with a tip-induced electrostatic AB effect associated with electron paths encircling an antidot in our sample.

However, interferences involving electron paths encircling smaller surfaces could lead to larger amplitude AB-like oscillations, which could be an alternative hypothesis explaining the origin of the anomalous phenomenon. Such oscillations should be visible in the magnetoconductance of the device, and their B-periodicity would allow to infer the area encompassed by interfering electron trajectories. To observe how the anomalous phenomenon evolves with the magnetic field, the derivative of *G* with respect to *V*_*tip*_, measured with the tip over the middle point of the central branch of the network, is mapped as a function of B in Fig. 5c - note that the central branch is not depleted by the tip, over the full *V*_*tip*_ range in Fig. 5c (more details about tip-induced depletion are provided in the Supplementary Figure S5). Strikingly, the anomalous blue domain observed between ${V}_{tip}\sim -8$ and −6 V persists over the full *B*-range, and its average position is only weakly modulated by *B* with an AB periodicity associated with trajectories around antidots (*i.e*. 7 mT). If an AB effect corresponding to a surface *S* trajectory was at the origin of the phenomenon, the sign of $\frac{{\rm{d}}G}{{\rm{d}}{V}_{tip}}|B$ should be reversed at constant *V*_*tip*_ with a periodicity corresponding to *ϕ*_0_/*S* (where *ϕ*_0_ is the quantum of flux). The absence of sign reversal in the *B*-range considered in Fig. 5c means that *S* is much smaller than *ϕ*_0_/30 mT = 0.068 *μm*^2^, which corresponds to a circle with a radius smaller than 148 nm. This radius is smaller than the antidot lateral size, but could fit into the central branch of the network. One could then imagine that the tip potential locally raises the potential in the central branch of the network, so as to create a small-area antidot which could generate AB-like oscillations with a large *B*-period. This hypothesis can be simply checked by putting the tip further away from the device. The anomalous behavior is now observed when varying *V*_*BG*_, as shown in Fig. 5b. The tip-induced antidot hypothesis is therefore ruled out. Note that the observation of the anomalous behavior for positive *V*_*tip*_ was anyway incompatible with this hypothesis. At that point, applying a magnetic field reveals that the anomalous phenomenon in $\frac{{\rm{d}}G}{{\rm{d}}{V}_{tip}}{\rm{v}}{\rm{s}}\,{V}_{BG}$ also remains essentially unaffected, up to 40 mT, as shown in Fig. 5d (although a small-amplitude 7 mT modulation also decorates the data). Since no sign reversal is observed for the anomalies over an even larger B-range than in Fig. 5c,d naturally leads to consider the possibility of coherent interferences between electron paths encompassing a vanishing surface and intrinsic to the 2DEG network, *i.e*. not induced by the tip.

**Figure 6:**
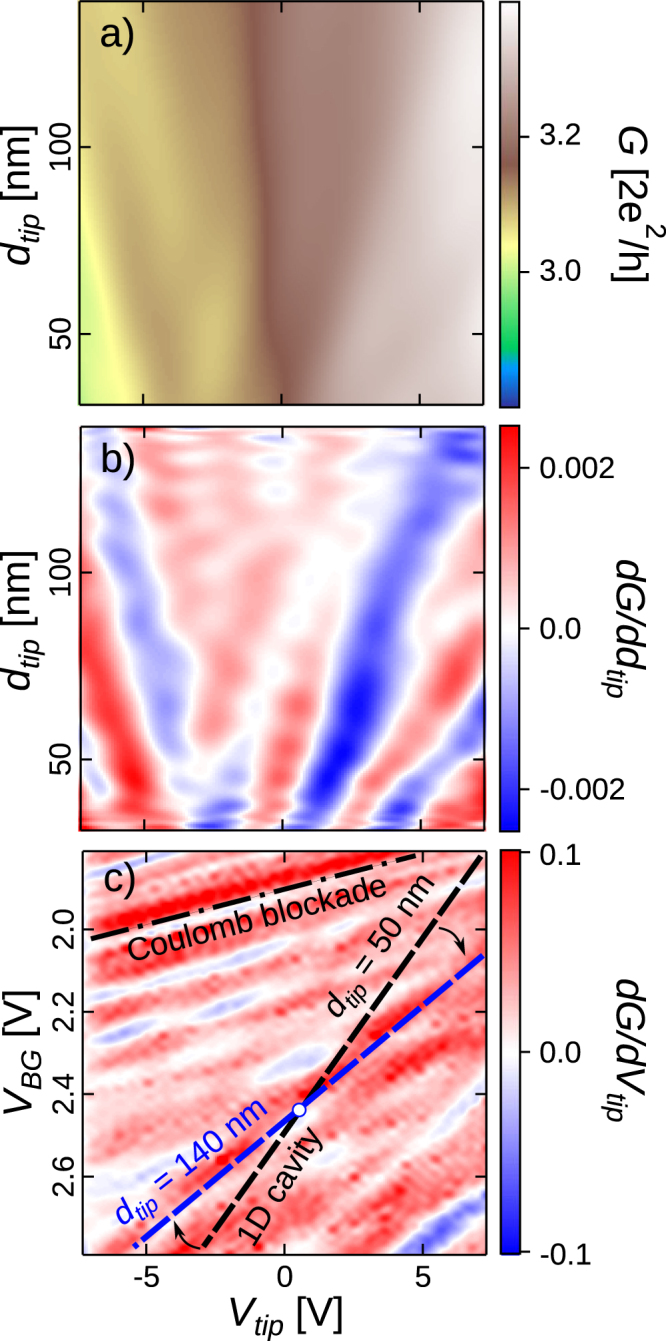
(**a**) Conductance measured for different values of *V*_*tip*_ and *d*_*tip*_ for the tip centered above the middle of the central branch (see Fig. 1a) *V*_*BG*_ = 3.8 V. (**b**) Derivative of *G* with respect to *d*_*tip*_ corresponding to the mapping (**a**). (**c**) Derivative of *G* with respect to *V*_*tip*_ at constant *V*_*BG*_ associated to the *G* mappings similar to Fig. 3b but realised for *d*_*tip*_ = 140 nm. The slope labelled ‘Coulomb blockade’ is identical to the slope 1 presented in Figs. 3c and 4c. We also reported the slope 2 identified on Fig. 3c obtained with *d*_*tip*_ = 50 nm (black dashed line) to compare with the slope observed with *d*_*tip*_ = 140 nm (blue dashed line). All horizontal axes are matched.

The latter idea corresponds indeed to the picture of standing electron wave patterns located within a 1D cavity inside the network. The tip-induced perturbation area is critical in this framework as it can tune the local electron density and hence change the interference pattern. A simple way to tune this parameter is to vary the tip-surface distance *d*_*tip*_ as shown on Fig. 6b, obtained with the tip remaining above the middle of the central branch. Oscillations of *G* and $\frac{{\rm{d}}G}{{\rm{d}}{d}_{tip}}|{V}_{tip}$ are visible as a function of both *d*_*tip*_ and *V*_*tip*_ in Fig. 6a and b, forming a fan-like structure. The observed convergence of the fringes on Fig. 6b at the level of the 2DES (${d}_{tip}\sim -45\,{\rm{nm}}$) indicates that they originate from a phenomenon triggered by the tip perturbation, occurring in the central branch right under the tip (cfr. Fig. 1c), as opposed to the CB-related fringes, associated with the effect of the tail of the tip perturbation (*i.e*. a long range effect). Note also the clear qualitative differences between Figs S2 and S3 which illustrate the very different effect of the tip in the CB and AB regimes.

**Figure 7:**
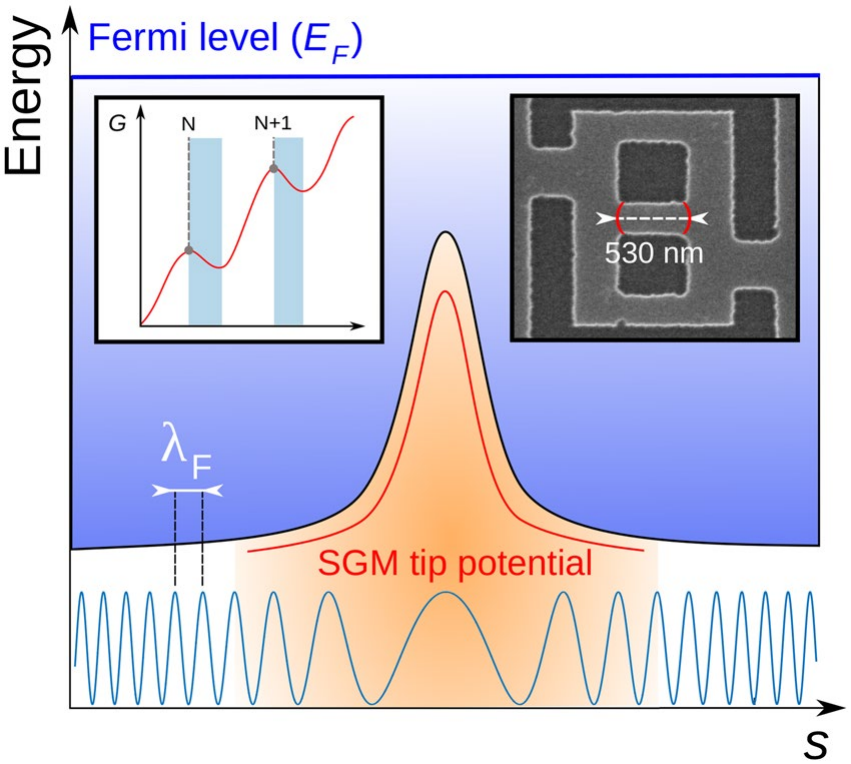
Illustration of how the SGM tip potential modifies locally the value of the bottom of the conduction band as the spatial variable *s* crosses the 1D cavity with the tip located above its middle. As the conduction band varies when the tip is polarised, it is possible to tune resonance conditions by locally modifying the electron wavelength. Insets: illustration of the *G* typical signature associated with successive resonance orders **N, N+1** etc; the estimated cavity length (530 nm) is reported on a network scanning electron micrograph.

The difference of behavior between both types of fringes with respect to changes of *d*_*tip*_ is further evidenced in $\frac{{\rm{d}}G}{{\rm{d}}{V}_{tip}}$ vs (*V*_*tip*_,*V*_*BG*_) maps (such data were already shown in Fig. 3c where *d*_*tip*_ = 50 nm). Such a map is presented in Fig. 6c with *d*_*tip*_ = 140 nm where both slope 1 and slope 2 - identified on Fig. 3c - are reported for comparison purposes. One can directly observe that the slope of fringes emerging at low *V*_*BG*_ (slope 1 fringes) does not vary with *d*_*tip*_, while slope 2 decreases as *d*_*tip*_ increases. A weaker slope means a larger *V*_*tip*_ period at constant *V*_*BG*_, which is consistent with Fig. 6b. The increasing period of the oscillations of $\frac{{\rm{d}}G}{{\rm{d}}{V}_{tip}}|{d}_{tip}$ vs *V*_*tip*_ when the tip goes away from the surface is compatible with the hypothesis of a local tip-induced variation of the interfering electron wavelength modifying resonant patterns in a 1D cavity^23,24^. This is illustrated in Fig. 7: in the area where the density is modified by the tip-induced perturbation, the Fermi wavelength changes according to $\sqrt{\frac{2\pi }{{n}_{2D}}}$. In turns this affects the cavity resonance conditions, defined by:

1 $${\rm{\Delta }}\varphi =\int ds\,{k}_{F}(s)=\int ds\sqrt{2\pi {n}_{2D}(s)}=N2\pi $$

where *k*(*s*) is the local Fermi wavevector, *s* the spatial variable along the cavity and *N* the resonance order.

In this framework, an oscillation of $\frac{{\rm{d}}G}{{\rm{d}}{V}_{tip}}|{d}_{tip}$ vs *V*_*tip*_ corresponds to a change of the resonance order *N* of the cavity. The increasing period of the oscillations observed when withdrawing the tip (Fig. 6b) is then explained by the diminishing lever arm of the tip over the local change in the Fermi wavelength. The *V*_*BG*_-period associated to these oscillations is roughly equal to 0.3 V, as shown on Fig. 5b. Considering the resonance hypothesis, each maximum is assumed to correspond to successive resonance orders *N*, *N* + 1, *N* + 2… At resonance, the following equation is relevant: 2*L*_*c*_ = *Nλ*_*F*_(*V*_*BG*_) where *L*_*c*_ is the cavity length and ${\lambda }_{F}=\sqrt{\frac{2\pi }{{n}_{2D}}}$ is the Fermi wavelength. One can therefore write:

2 $$\frac{N}{N+1}=\frac{{\lambda }_{F}^{N+1}}{{\lambda }_{F}^{N}}=\sqrt{\frac{{n}_{2D}^{N}}{{n}_{2D}^{N+1}}}$$

Since we measured *n*_2*D*_ as a function of *V*_*BG*_ (from SdH oscillations), we can determine *N* for the different resonances. For the maxima identified in Fig. 5b at *V*_*BG*_ = (3.1, 3.44, 3.8)[V], we have *n*_2*D*_ = (7.34, 7.74, 8.15)[10^15^ m^−2^], hence *λ*_*F*_ = (29.24, 28.48, 27.75)[nm]. The corresponding values of *N* are 36, 37, 38, which leads to a cavity length *L*_*c*_ of ~530 nm. Note that 2*L*_*c*_ is smaller than the elastic mean free path ${l}_{\mu }\sim 1.5\,\mu {\rm{m}}$ and comparable to the phase coherence length ${l}_{\varphi }\sim 1\,\mu {\rm{m}}$ - for *V*_*BG*_ = 3.8 V. *L*_*c*_ is larger than the central channel width so the 1D cavity is naturally located along the central branch, as schematically represented in Fig. 7 (white dashed line in the right inset). The edges of the cavity could correspond to the two open ends of the central branch: sharp changes of potential are expected at these locations, associated with the widening of the 1D channel, which could increase the reflection of electron wave functions into the 1D channel. However, further work is needed to confirm this hypothesis.

Now that we identified a clear *d*_*tip*_-dependency of the resonance, one can naturally wonder why slope 2 remains roughly unchanged in (*V*_*tip*_, *V*_*BG*_) mappings as *d*_*tip*_ goes from 50 to 100 nm (see Fig. 3c and f). To clarify this observation we realised a set of measurements in the same (*V*_*tip*_, *V*_*BG*_) conditions as those presented in Fig. 3b but for several values of *d*_*tip*_ between 50 and 140 nm. The interest of such *V*_*tip*_, *V*_*BG*_ maps is that the behavior of both slope 1 and slope 2 can be observed as *d*_*tip*_ changes. Qualitatively we would expect both slopes to decrease when rising *d*_*tip*_ - indeed no tip-induced conductance variation would be observed for a tip located at infinity. We are going to discuss both slopes separately. We observed that slope 1 remains unchanged no matter the value of *d*_*tip*_ between 50 and 140 nm (see Fig. 3c and Supplementary Figure S6). This is not surprising since the QDs leading to CB-related variations are located roughly at the center of the concentric fringes observed in Fig. 4f - *i.e*. a few hundreds of nanometers away from the center of the central channel. So, the charging events are almost insensitive to a change of 90 nm in *d*_*tip*_ and slope 1 is the same in Fig. 6c. On the contrary, as presented in Fig. 6c, slope 2 decreases when *d*_*tip*_ goes from 50 to 140 nm. A smaller slope means a higher *V*_*tip*_ period at constant *V*_*BG*_. This observation is consistent with Fig. 6b, where the iso-order resonance domains are going away from each other as *d*_*tip*_ increases. Also, slope 2 rotates around a particular point identified in Fig. 6c. Qualitatively, when the tip is brought away, one should apply a stronger tip potential in order to reach an equivalent resonant order. The domains located along slope 2 associated to the same resonance order should be rotating around a point where the tip is neutral with respect to the resonant pattern. It is possible to identify this particular point by checking where two equivalent domains for different *d*_*tip*_ are crossing. As expected, this point is located near *V*_*tip*_ = 0 V.

At the end of this discussion, we can now come back to the interpretation of the temperature dependence shown in Fig. 2. One question indeed naturally arises if we consider UCFs on one side, and the Fabry-Perot resonance mechanism that we identified in the central branch on the other side: since both phenomena stem from electron interferences, why do they exhibit different temperature dependences? To answer the question, another time scale should be considered, in addition to the phase coherence time: the electron dwell time^25,26^. The electron dwell time in the central branch constituting a 1D cavity ${{\rm{\tau }}}_{d}^{1D}$ is expected to be small compared to the dwell time in the whole device ${\tau }_{d}^{network}$. This has consequences on the temperature dependence of coherent oscillations. Indeed, as temperature decreases, the phase coherence time increases and a crossover is expected when it becomes larger than the cavity dwell time: below this crossover temperature - typically in the range 1–5 K for cavities similar to the ones considered here^21^ - the amplitude of coherent oscillations saturates. One can then understand the difference shown in Fig. 2a,b in the temperature dependence of the anomalous phenomenon associated to the 1D cavity characterized by a relatively small ${{\rm{\tau }}}_{d}^{1D}$, and of UCFs, related to interferences between semiclassical paths exploring the full device area (much larger than the 1D cavity). Figure 2a,b show that UCFs dominates at 50 mK, but undergo a fast decay when temperature rises so that the anomalous conductance phenomenon becomes visible at 4.2 K. One can therefore infer that the amplitude of the latter phenomenon saturates below 4.2 K, because ${\tau }_{\varphi } > {\tau }_{d}^{1D}$, while the UCFs amplitude increases below 4 K, because ${\tau }_{\varphi } < {\tau }_{d}^{network}$.

## Conclusion

We identified two mechanisms at the origin of the paradoxical increase of conductance observed in a mesoscopic network while gradually depleting one channel: charging CB events associated to disorder-induced localized states and resonance mechanism due to the presence of a 1D cavity in the central channel. The first phenomenon has already been extensively studied in previous works, and in particular in ref.^12^ in the context of open nanodevices (*G* > 2*e*^2^/*h*). Regarding the second mechanism, the electron wave resonance raises several questions and prospects. The formation of a 1D cavity is already puzzling: what causes electron wave reflections? Unlike resonant tunneling diodes, we revealed resonant state signatures in an open coherent and ballistic system where no tunneling mechanism is expected. Further work should allow to discriminate between different causes of reflection inside the structure (*e.g*. the abrupt variation of the electrostatic confining potential at the entrance of the 1D channel). By comparison with other channels in the network, only the central one could lead to a symmetric resonant cavity. In the top and bottom channels, one of their sides gradually curbs to form the right angle that connects them to the vertical channels, while the curvature of the etched sides is very sharp at the opening of the central channel. Is this the only reason why a resonance is only present in the central branch? We also stress out that the two mechanisms described in the current work are intrinsically different from the one evidenced in the simulations results from ref.^3^, where the anomalous behavior was observed on the verge of full depletion of the central channel. This means that paradoxical behaviors with different origins can be identified in nanoscale conducting networks.

## Electronic supplementary material

Supplementary figures
