# Supplementary material for: On the origins of transport inefficiencies in mesoscopic networks
Source: Sci Rep. 2018 Feb 14;8:3017. doi: 10.1038/s41598-018-21250-y (PMC5812991; doi:10.1038/s41598-018-21250-y)
Supplement: Supplementary file 1 — Supplementary figures [file 41598_2018_21250_MOESM1_ESM.pdf]

Supplementary Materials : On the origins of transport inefficiencies in mesoscopic networks

Sébastien Toussaint[1,*], Frederico Martins[1], Sébastien Faniel[1], Marco G. Pala[2], Ludovic Desplanque[3], Xavier Wallart[3], Hermann Sellier[4], Serge Huant[4], Vincent Bayot[1], and Benoit Hackens[1,*]

[1]Université catholique de Louvain, Institute of Condensed Matter and Nanosciences (IMCN/NAPS), Louvain-la-Neuve, B-1348, Belgium
[2]Centre de Nanosciences et de Nanotechnologies, Université Paris-Sud, Université Paris-Saclay, CNRS, Orsay, F-91405, France
[3]Université Lille, CNRS, Centrale Lille, ISEN, Univ. Valenciennes, UMR 8520 - IEMN, Lille, F-59000, France
[4]Institut Néel, Université Grenoble Alpes and CNRS, Grenoble, F-38042, France
[*]sebastien.toussaint@uclouvain.be, benoit.hackens@uclouvain.be

ABSTRACT

A counter-intuitive behavior analogous to the Braess paradox is encountered in a two-terminal mesoscopic network patterned in a two-dimensional electron system (2DES). Decreasing locally the electron density of one channel of the network paradoxically leads to an increased network electrical conductance. Our low temperature scanning gate microscopy experiments reveal different occurrences of such puzzling conductance variations, thanks to tip-induced localized modifications of electron flow throughout the network's channels in the ballistic and coherent regime of transport. The robustness of the puzzling behavior is inspected by varying the global 2DES density, magnetic field and the tip-surface distance. Depending on the overall 2DES density, we show that either Coulomb Blockade resonances due to disorder-induced localized states or Fabry-Perot interferences tuned by the tip-induced electrostatic perturbation are at the origin of transport inefficiencies in the network, which are lifted when gradually closing one channel of the network with the tip.

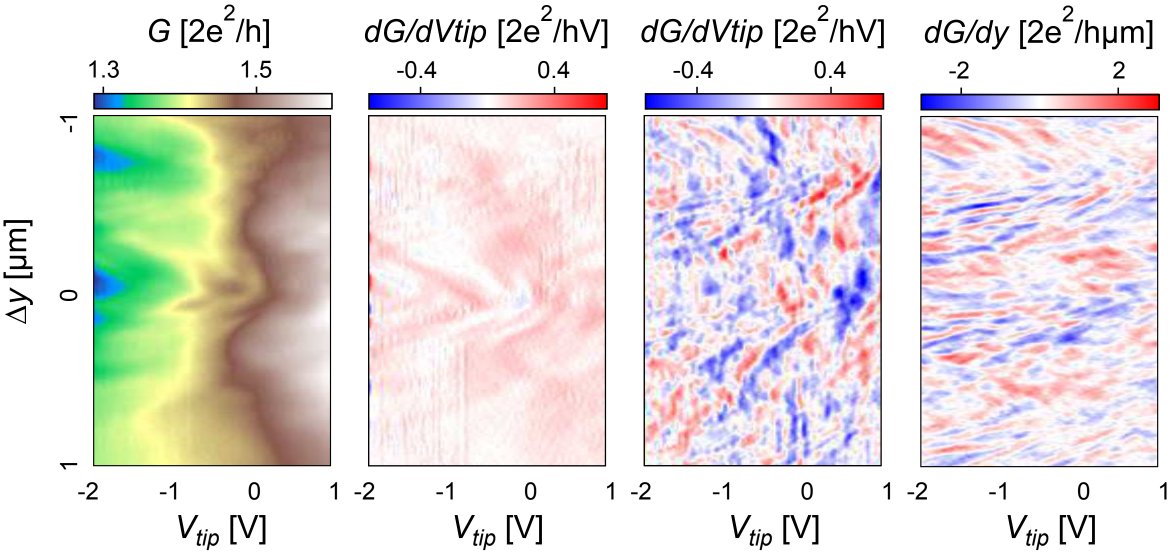

Figure 1. a) Conductance measurement as a function of V_{tip} with the tip scanned along the dashed white line seen in Fig.1a for d_{tip} = 80 nm and V_{BG}= -0.1 V. b) Derivative of G with respect to V_{tip} of the data presented in (a). c) Derivative of G with respect to V_{tip} of the data presented in Fig.2b. d) Derivative of G with respect to y of the data presented in Fig.2b. All vertical axes are matched.

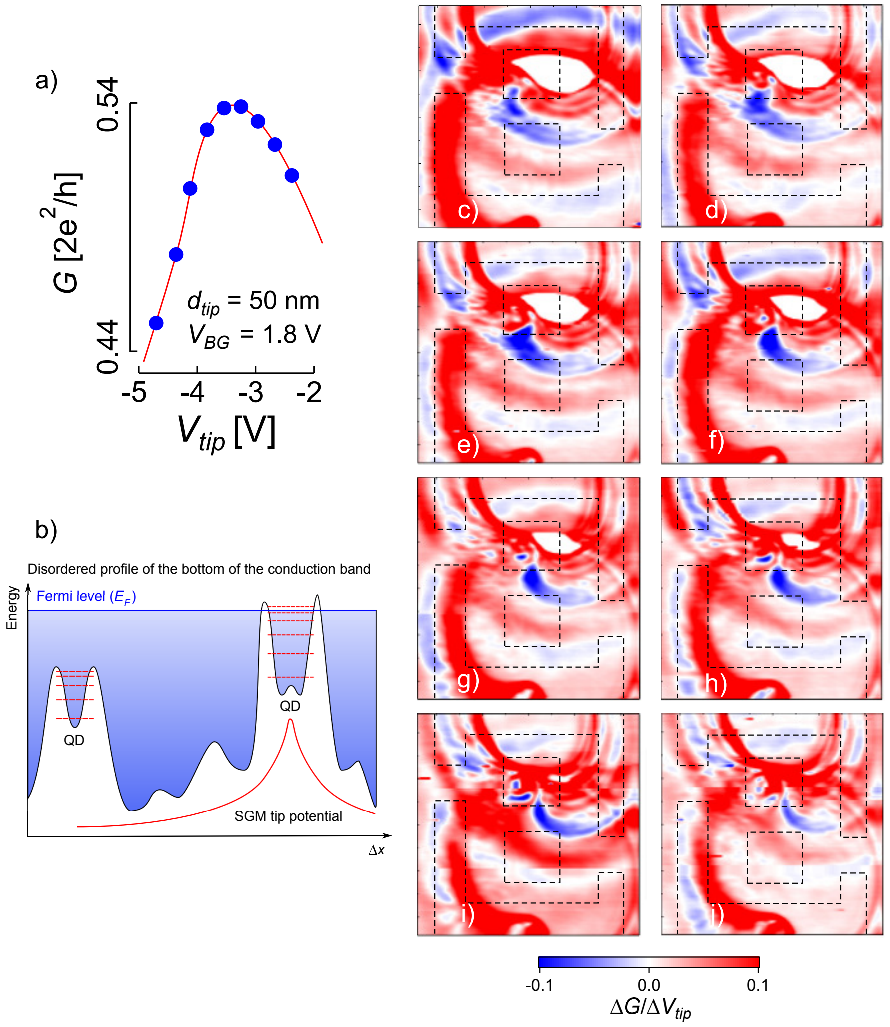

Figure 2. (a) Conductance vs V_{tip}, measured at V_{BG}= 1.8 V and d_{tip}= 50 nm, with the tip positioned above the middle of the central arm. The blue dots indicate the tip voltages where SGM mappings were realized. (b) Schematic picture illustrating the effect of the SGM tip potential (red continuous line) on the bottom of the conduction band (black line). The tip potential raises disorder-induced fluctuations up to the Fermi level. This creates a confined quantum dot structure, tunnel-coupled to the leads, with discrete energy levels brought in resonance with the Fermi energy for specific tip position and voltage. (c-j) Differential SGM mappings, obtained by subtracting conductance maps measured at two consecutive V_{tip} polarizations indicated by the blue dots in (a). Shrinking concentric fringes are observed, directly related to the tip-controlled tuning of energy levels inside quantum dots located within the device. The sequence of red-white-blue fringes crossing the central arm as V_{tip} is varied in (c-j) allows to deduce that the conductance maximum in (a) is directly related to the Coulomb blockade mechanism depicted in (b).

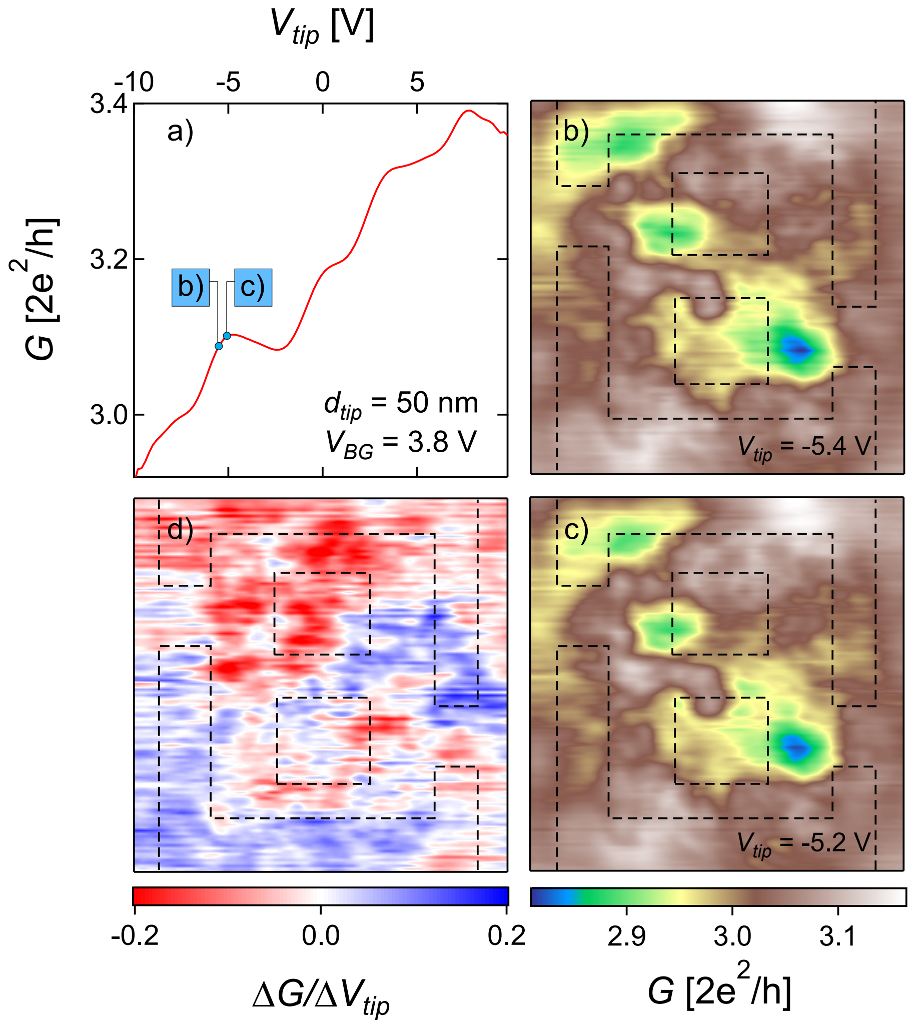

Figure 3. (a) Conductance vs V_{tip} with the tip located 50 nm above the middle of the central channel and V_{BG}=3.8 V. (b) and (c) SGM conductance maps of the device at V_{tip}=-5.4 and -5.2 V, with d_{tip}=50 nm and V_{BG}=3.8 V. (d) Subtraction of the two SGM SGM maps shown in (b) and (c).

.

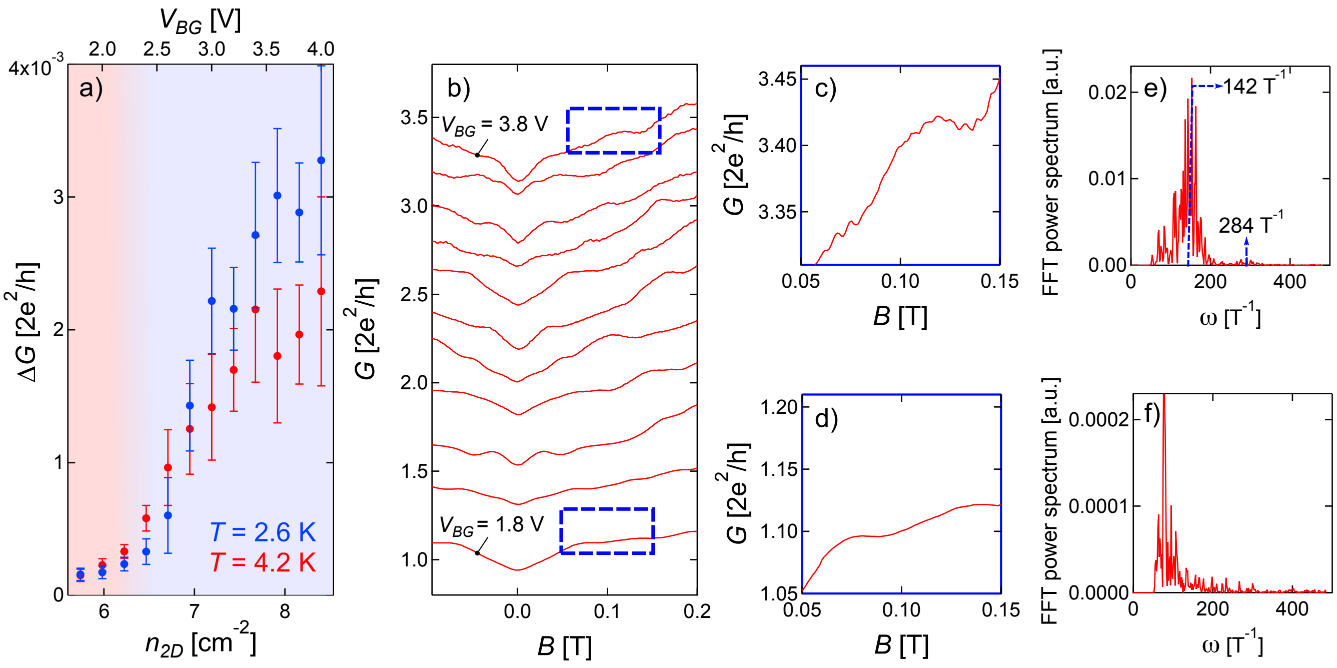

Figure 4. (a) RMS amplitude of the AB oscillations for different values of V_{BG} - *i.e.* different n_{2D} - at $T = 2.6$ K and $T = 4.2$ K. (b) G vs B measured for different values of V_{BG} between 1.8 and 3.8 V. (c), resp. (d), gives an enlarged view of a small range of magneto-conductance curves obtained at V_{BG}=3.8 V, resp. 1.8 V [blue dashed boxes shown in (b)]. (e), resp. (f), FFT of the magnetoconductance curves displayed in (c), resp. (d). Note that magnetoconductance curves were high - pass filtered. The FFT presented in (e) exhibits maxima at 142 and 284 T^{-1}, corresponding to 7 mT and 3.5 mT AB periods. The 142 T^{-1} maximum is consistent with orbits enclosing a single antidot in the device. The peak at 284 T^{-1} could correspond either to Altshuler-Aronov-Spivak (AAS) oscillations, or to AB oscillations associated with orbits enclosing both antidots. We favour the AAS hypothesis since the 284 T^{-1} peak disappears when time-reversal symmetry is lifted as B grows. None of the above-mentioned maxima could be identified in (f).

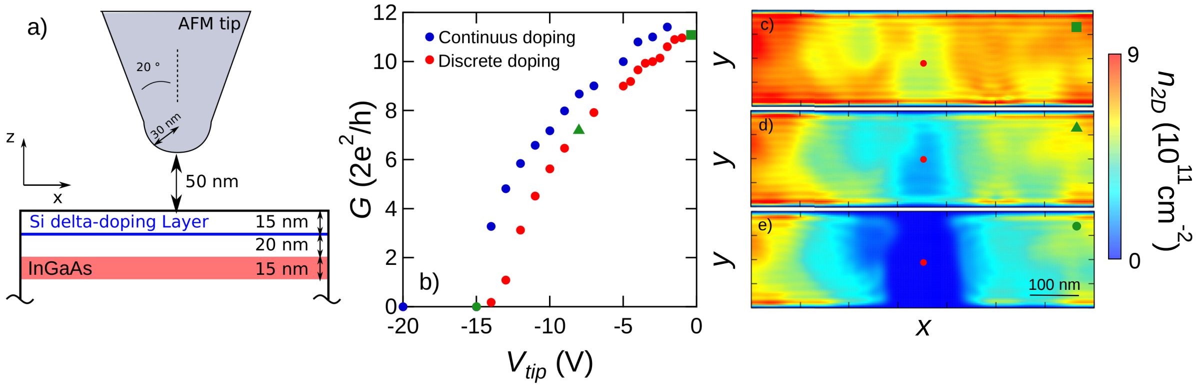

Figure 5. (a) Schematic side-view of the tip geometry and its position with respect to the 2DES, located in the InGaAs layer in red. (b) Simulated conductance vs V_{tip} in a 200 nm-wide channel patterned in an InGaAs/InAlAs heterostructure, mimicking the central channel in the mesoscopic network discussed in the main text, with random dopants inducing disorder in the potential (red data points), or with homogeneous continuous doping (blue data points). We used the same doping concentration as in the experiment. These calculations are based on three-dimensional self-consistent simulations coupling the Poisson equation in the whole volume of Fig. 5(a) and the Keldysh-Green function equations in the wire region [M. G. Pala et al., Phys. Rev. Lett. 108, 076802 (2012)]. This method allows us to accurately simulate the tip-induced potential experienced by conduction electrons. Noteworthy, depletion occurs for $V_{tip} < -14$V. (c-e) Real-space mapping of the electron density within the 200 nm-wide channel with random dopants distribution, for V_{tip} = -0.5, -8 and -15 V, respectively - the tip position is given by the red dot in each figure. These images give a direct view of the extension of the tip perturbation inside a channel similar to the central channel of the mesoscopic network studied in the main text.

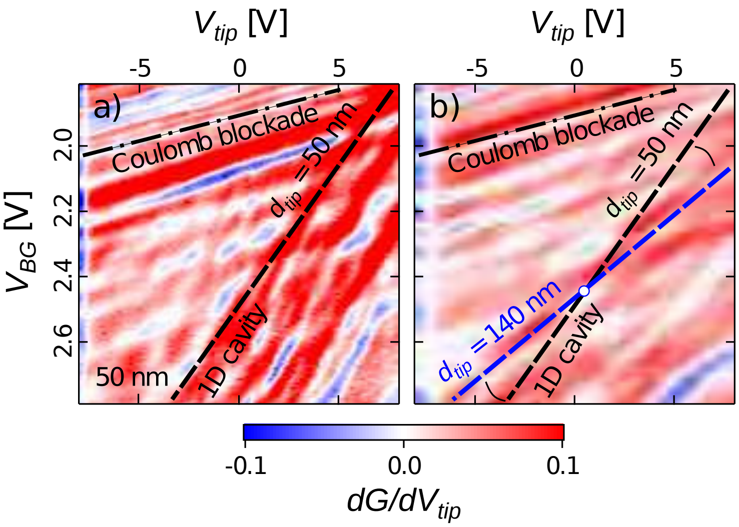

Figure 6. (a) and (b) present the derivative of G with respect to V_{tip}, with the tip located above the middle of the central branch, at d_{tip}= 50 nm (a) and 140 nm (b).
